# Interrelationships Between Ki67, HER2/neu, p53, ER, and PR Status and Their Associations With Tumor Grade and Lymph Node Involvement in Breast Carcinoma Subtypes: Retrospective-Observational Analytical Study

**DOI:** 10.1097/MD.0000000000001359

**Published:** 2015-08-14

**Authors:** Taghipour Zahir Shokouh, Aalipour Ezatollah, Poorya Barand

**Affiliations:** From the Department of Clinical and Surgical Pathology (TZS); and Student Research Committee (AE, PB), Shahid Sadoughi University of Medical Sciences, Yazd, Iran.

## Abstract

Various predictive and prognostic factors could affect breast carcinoma behavior, but to date no definitive correlation has been established between them and breast carcinoma subtypes. The present study was conducted to examine the interrelationships of these predictive and prognostic factors as well as their effects on breast carcinoma subtypes.

The archives of all patients with breast carcinoma (from 2008 to 2014) were studied. Patients’ data were extracted using a checklist that included age, histology type, size and grade of tumor, lymph node involvement, estrogen receptor (ER) and progesterone receptor (PR) status, along with the overexpression of human epidermal growth factor receptor (HER2/neu) and the rate of Ki67 and p53 mutations. All data were analyzed by SPSS-17 software with χ^2^ and Fisher exact tests, as well as the least significant difference pairwise comparison test.

A total of 566 patients’ records were included in this study. The mean age of patients was 50 ± 12.9 with an age range of 17 to 98 years. A meaningful correlation was found between age and the type of tumor (*P* = 0.001). Infiltrating lobular carcinoma had a higher ER positivity between groups (85.7%), whereas noninvasive carcinomas had a higher PR positivity (67%). In addition, a meaningful correlation was detected between the type and grade of tumor (*P* = 0.001). No meaningful relationship was observed between the type of tumor and HER2/neu overexpression and number of lymph nodes involved. Between the groups, medullary carcinoma had the highest Ki67 index (*P* = 0.007). Meaningful correlation was found between the grade of tumor and lymph node involvement (*P* = 0.005) and also with HER2/neu overexpression (*P* = 0.002). Higher grades had greater positivity in Ki67 index and p53 mutation rates (*P* = 0.002, *P* = 0.01). HER2/neu positive tumors had a higher Ki67 index (*P* = 0.03).

Higher Ki67 index tumors showed more HER2/neu overexpression, larger size, and more lymph node involvement compared with other types and maybe considered aggressive. Moreover, in young patients with breast carcinoma, the rates of Ki67 with the overexpression of HER2/neu and p53 mutations are higher, and it shows a more aggressive behavior than other tumors assessed in this age group.

## INTRODUCTION

Breast cancer is the most common cancer type among women. The mortality rate has been significantly reduced in recent years because of its early diagnosis and the advanced methods of treatment; however, it is still the second leading cause of death from cancer in women in European and Western countries, preceded only by lung cancer.^1,2^ In Iran, breast cancer is the fifth leading cause of death from cancer; it affects 1 in every 8 women and has an annual incidence rate of 31 per 100,000 women, meaning that 8500 individuals are diagnosed with breast cancer each year. In Iran in the last 5 years, 30,000 women died from breast cancer.^3^ By using screening methods such as ultrasound and mammography, and by continuously training women to conduct self-examination, the age of breast cancer incidence has reduced in the last few years from the fourth decade of life to the second and third decades.^3^ Various predictive and prognostic factors affect tumor progression.^4–6^ Predictive factors are distinguished from prognostic factors in that the latter can be measured and are associated with the nature of the disease, whereas the former determine the response to treatments.^4^ Some factors are both prognostic and predictive, including estrogen receptor (ER) and progesterone receptor (PR) status, p53 mutation status, and human epidermal growth factor receptor (HER2/neu) overexpression. Prognostic factors include the type of tumor, number of involved lymph nodes at the time of tumor diagnosis, size of the tumor, tumor grade, Ki67 status (cellular marker for proliferation), and the patient's age.^5,7^ Numerous studies have been conducted on these prognostic factors and their relationships with one another; however, the studies have reported disparate results.^4–7^ Therefore, the present study was conducted to analyze cases of breast cancer in a university medical center in an effort to find a logical relationship between the type of tumor, and these predictive and prognostic factors perhaps identify a better diagnostic method that increases the patients’ longevity and improves their quality of life.

## METHODS

The present retrospective-observational analytical study was conducted on all female breast cancer patients admitted to the pathology department of Shahid Sadoughi Hospital of Yazd in Iran between 2008 and 2014. Shahid Sadoughi Hospital is the main university hospital and referral center in Yazd for immunohistochemical testing of cancer tumors. The research deputy of the medical school of Shahid Sadoughi University of Medical Sciences approved this study. Informed consent was obtained from all individual participants included in the study. Our exclusion criterion was metastatic carcinomas to the breast. A total of 566 cases were found eligible and enrolled in the current study, and 13 patients with metastatic tumors to the breast were excluded. The patients’ information was extracted from their pathology and hospital records according to the prepared checklist and considering the predictive and prognostic variables such as age, tumor size, histologic subtype and grade of tumor, number of involved lymph nodes, p53 mutation status, ER and PR status, HER2/neu overexpression, and Ki67 labeling index. The patients’ pathological slides were extracted from the archives, reviewed again separately by 2 qualified pathologists, each blinded to the participation of the other. Breast carcinoma was divided into 2 major groups based on a WHO classification of breast carcinoma: invasive and noninvasive. The invasive group was divided into the ductal, lobular, tubular, mucinous, and medullary categories. The rare types, including the secretory, apocrine, and neuroendocrine types, were classified under the “others” category because of their small number. The Scarff-Bloom-Richardson standard grading system was used to grade the tumors. The tumors were graded as 1, 2, or 3 based on the formation or nonformation of tubules, their nuclear pleomorphism, and the number of mitosis. Grade 1 is defined as a well-differentiated tumor with the best prognosis, grade 2 is a moderately differentiated tumor with an intermediate prognosis, and grade 3 is a poorly differentiated tumor with the worst prognosis. The slides were previously immunohistochemically stained according to the manufacturer's guidelines (Dako, Glostrup, Denmark), and were retrieved from the archives and reviewed separately by 2 pathologists. To estimate the percentage of cells that stained positive for ER and PR, 100 tumor cells were counted, and the ratio of the number of stained cells to the total number of cells was calculated and reported as a percentage. The intensity of reaction was graded as negative, weak, moderate, or strong. Both parameters (percentage and intensity) were used in scoring system of ER and PR status. The tumor cells with >20% stained were considered as positive, those with 5% to 19% stained were considered as borderline, and those with <5% stained were designated negative; however, in the final report, all the tumor cells with >5% stained were considered positive. For the HER2/neu overexpression, the data were classified from 0 to 3 based on the criteria provided by Dako (Glostrup); the scores 0 and 1+ were considered as negative, 2+ as borderline, and 3+ as positive. All the cases with a 2+ score were sent to another center for the FISH test, and all the cases with a positive FISH were identified as HER2/neu overexpression positive. To report p53 mutation status based on the slides immunohistochemically stained according to the guidelines of Dako (Glostrup), the tumor cells with >10% stained were considered as positive, and those with <10% stained considered as negative. The Ki67 immunohistochemically stained slides for Ki67 marker were divided into 4 groups: the nonstained as negative, those stained up to 10% as low, those stained over 10% to 20% as borderline, and >20% as high; however, the cells stained over 10% were considered as positive. Overall, breast carcinomas were divided into 4 major molecular subtypes: Luminal A (ER+ and/or PR+, HER2−, low Ki67), Luminal B (ER+ and/or PR+, HER2+ or HER2− with high Ki67), Triple negative/basal-like (ER−, PR−, HER2−), and HER2 type (ER−, PR−, HER2+).^8^

### Statistical Tests and Analyses

Statistical analyses were performed using SPSS software version 17 for windows (IBM Inc, NY). To determine the relationship between qualitative variables, χ^2^ test was used; in case of a small sample size, however, we used Fisher exact test. Therefore, for testing the quantitative variables, *t* test and analysis of variance (ANOVA) as well as least significant difference (LSD) test for pairwise comparison were used. Continuous data (such as age, lymph node involvement, and tumor size) between groups were expressed as mean ± standard deviation (SD) and categorical data as frequency counts (percentages). All qualitative data (ER, PR status, HER2/neu overexpression, p53 mutation status, and Ki67 index) based on breast cancer subtypes were analyzed by χ^2^ test. To compare pairs in groups, LSD test was used. χ^2^ test was used for the comparison of the frequency of lymph node involvement between groups, and ANOVA was used for the comparison of the mean of Ki67 and p53 mutation in different HER2/neu overexpression groups. We then used LSD pairwise comparison test on the basis of significances of Ki67 in HER2/neu groups. *P* values <0.05 were considered statistically significant.

## RESULTS

The present study was conducted on 566 breast cancer patients with a mean age of 50 ± 12.9 years and an age range of 17 to 98 years. Invasive ductal carcinoma 328 (58%) and carcinoma in situ 136 (24%) were the more prevalent cases of breast cancer. The least prevalent case was mucinous carcinoma, with a frequency of 9 (16%). In terms of tumor grades, grade 2 was the most prevalent (195; 54.8%) and grade 1 was the least prevalent (35; 1.8%). The details of lymph node involvement were available only for 423 patients, with 251 (59.3%) to have their lymph nodes involved. ER details were available in 523 patients, showing 336 (64.2%) of tumors to have this type of receptor, and PR details were available in 521 patients, showing 297 (57%) of tumors to have this type of receptor. There was a significant correlation between the type of breast cancer and ER status, and medullary carcinomas showed the lowest frequency of ER expression (5; 19.2%) and lobular carcinomas the highest (24; 85.7%) (χ^2^ test, *P* = 0.001). Analysis of the relationship between PR status and the type of breast cancer showed that PR expression was significantly correlated with the type of breast cancer. Noninvasive carcinomas and tubular carcinoma (67% and 66.7%) had the highest frequency of PR expression (χ^2^ test, *P* = 0.001), whereas the lowest frequency of PR expression pertained to medullary carcinomas (3; 11.5%). HER2/neu overexpression was examined in 514 patients, with 182 (35.4%) of the tumors demonstrating HER2/neu overexpression (Table 1). Invasive ductal and in situ carcinomas had the highest frequency of HER2/neu overexpression (35.4% and 34.9%, respectively); however, no significant relationship was observed between the type of cancer and HER2/neu overexpression (Fisher exact test, *P* = 0.63). Analysis of the frequency of tumor grades showed the most prevalent type to be grade 2 (195; 54.8%), and a significant relationship was established between tumor grade and the type of cancer. Invasive lobular carcinoma was found to be the most prevalent grade 2 tumor (6; 60%), whereas medullary carcinoma was the most prevalent grade 3 tumor (2; 66.7%) (Fisher exact test, *P* = 0.001). Analyses showed no significant relationship between the type of tumor and lymph node involvement (*P* = 0.056), although invasive lobular carcinoma was shown to have the highest frequency of lymph node involvement (14; 60.9%) whereas mucinous and tubular carcinomas showed the lowest frequency of lymph node involvement (33.3% and 25%, respectively). As shown in Table 2, a significant relationship was found between the type of tumor and the age of incidence, such as medullary carcinoma, which was not the case in patients over the age of 70 (χ^2^ test, *P* = 0.001). A significant relationship was found between the type of tumor and the Ki67 index; therefore, medullary carcinoma had the highest frequency of Ki67 expression (17; mean = 55.9). The mean size of tumors was 2.9 cm, and the largest tumor size belonged to the lobular carcinoma (mean 3.5 cm), whereas tubular carcinoma had the smallest size (mean 1.9 cm); however, no significant differences were found between the size of tumor and its type (ANOVA, *P* = 0.74). LSD pairwise comparisons did not show any significant relationship between age and lymph node involvement (*t* test, *P* = 0.076). The pairwise comparisons did not show any significant relation between age and ER expression, age and PR expression, or age and HER2/neu overexpression (*P* was calculated as 0.059, 0.94, and 0.37, respectively). Analyses showed grade 3 tumors demonstrated the highest frequency (72; 73.5%) of lymph node involvement, suggesting a significant relationship between tumor grade and lymph node involvement (*P* = 0.005), so that the relative frequency of lymph node involvement increased with tumor grades. The frequency of HER2/neu positive (3+) breast carcinoma, triple negative breast cancers, Luminal A (ER+, PR+, HER2/neu−, Ki67 < 14%) breast cancers, and Luminal B (ER+, PR+, HER2/neu−, Ki67 > 14%) breast carcinomas were calculated, and the results are shown in Table 1. Ki67 expression by triple negative type and HER2/neu positive type breast carcinomas was analyzed, and significant difference was noted between these groups (Table 3). In addition, there was a significant correlation between tumor grade and HER2/neu overexpression, and HER2/neu was more frequently expressed with higher tumor grades (*P* = 0.02). Analysis of the relationship between Ki67 and tumor grade showed that Ki67 increased with tumor grade, and grade 3 tumors had the highest mean Ki67 value (45.8), whereas grade 1 tumors had the lowest (23.2); (ANOVA, *P* = 0.001; Table 4). p53 expression increased with tumor grade; grade 1 tumors had a mean of 7.7 and grade 3 tumors had a mean of 27.16, so the relationship between these 2 parameters was statistically significant (ANOVA, *P* = 0.014; Table 4). In the LSD pairwise comparisons, p53-positive tumors were reported to have less lymph node involvement (mean p53 = 17.5), whereas p53-negative tumors were reported to have more lymph node involvement (mean p53 = 24); however, there was no significant relationship between p53 mutation status and lymph node involvement (*P* = 0.17). Nevertheless, a significant relationship was found between the size of tumor and lymph node involvement, as lymph node involvement increased with the size of tumor (ANOVA, *P* = 0.003). The mean size of tumors with lymph node involvement was 3.28 cm, whereas the mean size of tumors without lymph node involvement was 2.58 cm, indicating a significant difference (*t* test, *P* = 0.001). Pairwise comparison showed a significant relationship between HER2/neu overexpression and Ki67 labeling index, as HER2/neu overexpression increased with Ki67 index (*P* = 0.03). However, no significant relationship was found between p53 mutation status and HER2/neu overexpression (*P* = .12) (Table 5). The correlation coefficient between both Ki67 index and p53 mutation and the size of tumor and age was calculated using Pearson correlation test, which showed that the correlations between the size of tumor and Ki67 expression (*r* = 0.139 and *P* = 0.02) and between age and Ki67 expression (*r* = −0.181 and *P* = 0.001) were significant, whereas the correlations with p53mutation were not significant (*r* = 0.91 and *P* = 0.19) (Table 6). Likewise, the correlations of p53 with the size of tumor and age were not significant (*r* = −0.084, *P* = 0.28 and *r* = −0.064, *P* = 0.35, respectively). There was an inverse correlation between age and p53 mutation, but this correlation was not statistically significant (Table 6). Analysis of the relationship between molecular subtype and age showed that no significant relationship was found between Luminal A subtype and patient's age (*P* = 0. 41, Fisher exact test); however, this type was more frequently expressed in patients <50 years (76; 57.6%) compared with patients >50 years (56; 42.4%). No significant correlation was found between triple negative and HER2 type with age (*P* = 0.21 and *P* = 0.48, respectively, Fisher exact test).

**TABLE 1:**
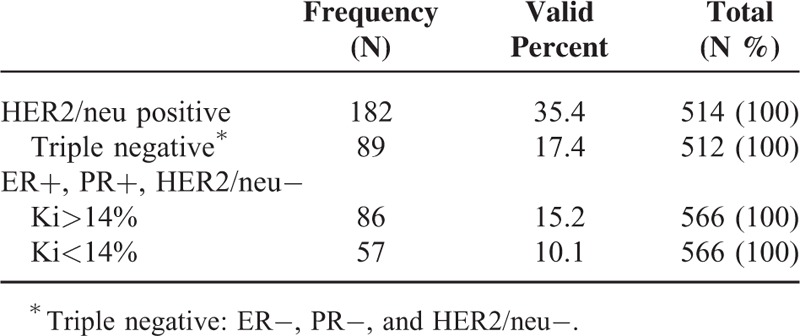
Frequency Distribution of Molecular Subtypes

**TABLE 2:**
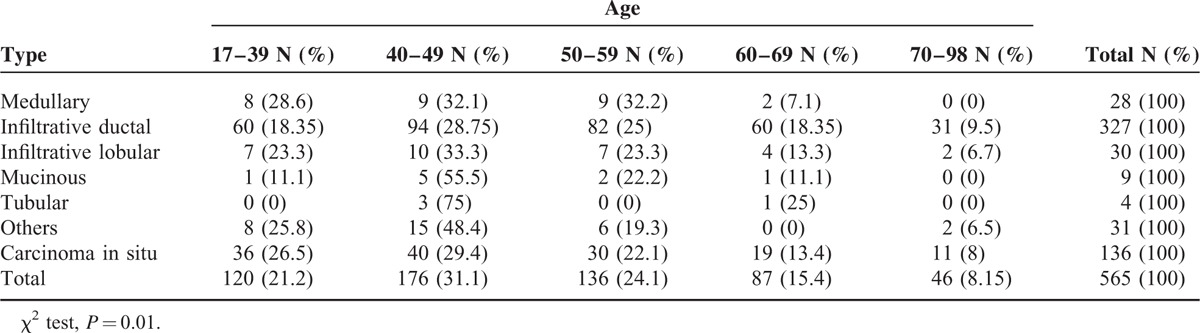
Frequency Distribution of Different Types of Breast Cancer Based on Age

**TABLE 3:**
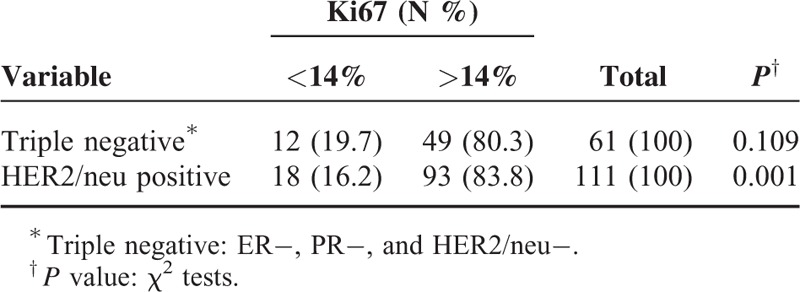
Ki67 Expression by Triple Negative Type and HER2/neu Positive Type Breast Cancer

**TABLE 4:**
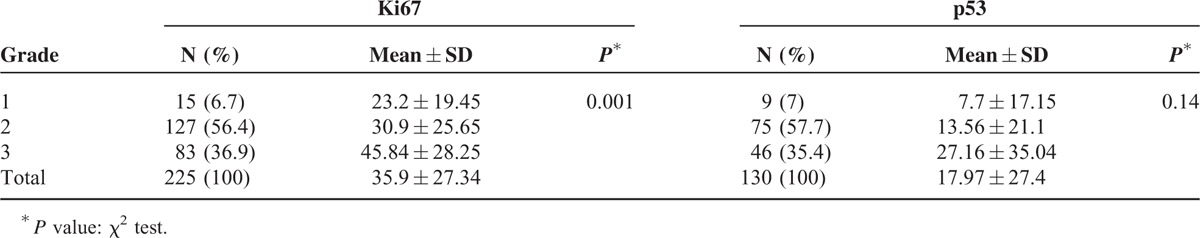
Correlation of Tumor Grade With Ki67 Index and p53 Mutation Rates

**TABLE 5:**
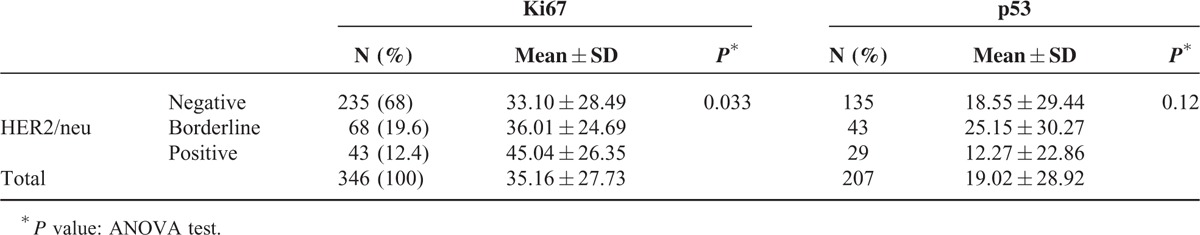
Correlation Between HER2/neu Status With Ki67 Index and p53 Mutation

**TABLE 6:**
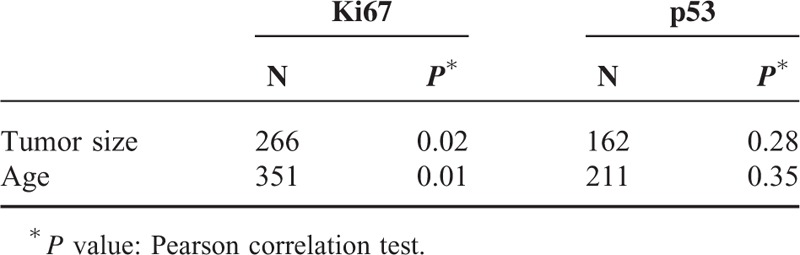
Correlation Between Tumor Size, Age With Ki67 Index, and p53 Mutation

## DISCUSSION

Breast cancer is one of the most prevalent cancers in women.^6^ Various factors affect the prognosis and relapse of this disease; each type of breast cancer exhibits distinct behavior, and whereas some types of breast cancer metastasize quickly, others are slow to metastasize and for a few, distant metastasis never occurs. The present study was therefore conducted to examine the status of different prognostic and predictive factors in breast carcinoma subtypes and to elucidate the interrelationships between these factors in order to identify significant relationships between the factors themselves and between the factors and the breast cancer types. One of the prognostic factors is breast carcinoma histologic subtype. Invasive ductal carcinoma and carcinoma in situ are the most prevalent type of breast carcinomas.^7,9^ In our study similar to other studies, the most common type of breast carcinoma was invasive ductal carcinoma.^1–3^ In a statistical study conducted by the UK cancer registry, breast cancer had 2 age peaks (1 in the 50–59 age group and the other in the 65–70 age group), but stabilized for a period before increasing again from age 75.^2^ However, in our study, the majority of patients with breast cancer were 1 decade younger than European countries.^2^ In a study conducted in India, cases of breast cancer were mostly younger than age 50 and it therefore appears that breast cancer patients are younger in Asian countries than in European countries.^11^ Studies conducted in other countries reported the prevalence of carcinoma in situ as 15% to 20 %; the present study reported the prevalence as 24%, which is in concordance with preceding reports.^7,9^

ERs and PRs are both predictive and prognostic factors and play a crucial role in the treatment of patients with breast cancer.^12,13^ On the basis of the current study, invasive lobular carcinoma had the highest frequency of ER expression, followed by noninvasive tumors. As a result, although invasive ductal carcinomas were more frequent, they behaved differently in terms of their ER status, and those 2 carcinomas (lobular and ductal) appear to respond better to hormone therapy. The lowest frequency of ER and PR expression is related to the medullary carcinomas, which is consistent with findings of other studies.^4,14^ In a study conducted by Li et al^15^ on the hormonal status of patients with breast cancer, the highest frequency of ER expression pertained to mucinous and invasive lobular carcinomas, which was inconsistent with the findings of the present study in which mucinous carcinomas had a lower frequency of ER expression compared with lobular carcinomas. In our study, carcinoma in situ had the highest and medullary carcinoma the lowest frequency of PR expression, which might suggest that in situ carcinomas progress very slowly under hormone suppression therapy and it might even be possible to prevent them from becoming invasive over time.^4^ In a study conducted by Bae et al,^16^ however, the highest frequency of PR expression pertained to mucinous carcinoma. On the basis of literature review, mucinous or colloid carcinomas metastasize later and are less likely to involve the lymph nodes at the emergence of the tumor.^17^ Although 30% to 40% of medullary carcinomas were PR positive in the cited study,^18^ the present study found only 11.5% of medullary carcinomas to express PR, suggesting that the medullary cancer patients examined in the present study are less likely to benefit from hormone suppression therapy. HER2 receptors are on breast cells and normally control their growth, division, and repair. In approximately 25% of breast cancers, the HER2 gene amplified and the result is HER2/neu overexpression that causes uncontrolled growth and division of breast cells.^15–17^ Almost all of the high-grade in situ breast carcinomas have a HER2/neu overexpression, but lobular carcinomas are less likely to demonstrate overexpression of HER2/neu.^18^ In the present study, our patients had greater HER2/neu overexpression compared with other studies as well as the highest frequency of HER2/neu overexpression related to invasive ductal carcinomas and the lowest frequency related to mucinous carcinomas, suggesting the higher invasiveness and greater aggressiveness of ductal carcinoma compared with other types. On the basis of this study, it appears likely that invasive ductal carcinomas have a greater likelihood of lymph node involvement compared with other types; however, this relationship was not statistically significant, and our study is in line with the previous published studies.^19^ Following invasive ductal carcinomas, carcinoma in situ had the highest frequency of HER2/neu overexpression. In a study conducted by Latta et al, 50% to 60% of noninvasive carcinomas had HER2/neu overexpression, and given that HER2/neu overexpression in this group is associated with a higher prevalence of local relapse.^20^ In our study, cases of breast cancer with HER2/neu overexpression were mostly younger than age 50; however, no significant relationships were found between the 2 parameters. The present study was consistent with other studies in that HER2/neu overexpression was mainly observed in patients <50.^21,22^ In the present study, HER2/neu overexpression increases with tumor grade, suggesting the higher likelihood of HER2/neu expression at higher grade tumors, thereby causing the tumors to be more invasive and more likely to relapse. Similar studies also showed the increase in HER2/neu overexpression with tumor grade.^22,5^

Another prognostic factor of breast cancer is tumor grade; higher tumor grades are associated with an increased degree of relapse, greater extent of involvement, and higher chance of distant metastasis.^23,24^ In the present study, grade 2 tumors were more prevalent than the other tumor grades (1 and 3), and lobular carcinomas had the highest frequency of grade 2 tumors, followed by invasive ductal carcinomas. The comparison of the different types of carcinomas showed that medullary carcinomas had the highest frequency of grade 3 tumors, and although high grade in appearance, this carcinoma acts as a low-grade tumor that does not progress rapidly and involves regional lymph nodes to a lesser degree.^25^ The present study showed a significant relationship between tumor grade and the extent of lymph node involvement. The highest levels of lymph node involvement were observed in grade 3 carcinomas. Moreover, the present study showed that lobular carcinomas were expressed with higher levels of lymph node involvement, followed by invasive ductal carcinomas; and although medullary carcinomas had a higher grade, their level of lymph node involvement was lower compared with lobular and ductal carcinomas; however, the difference was not statistically significant. Among all types of carcinomas, tubular carcinomas had the optimum grade (grade 1) and the lowest level of lymph node involvement and are therefore accompanied by the best prognosis and the least level of distant metastasis and local relapse.^25^ Ki67 was considered as a prognostic factor; in literature review high index labeled Ki67 is considered an unfavorable factor that influences tumor progression and is associated with poorer prognosis.^26^ In the present study, medullary carcinomas had the highest rate of Ki67 expression, followed by mucinous carcinomas, although both these carcinomas showed a lower degree of local relapse and lymph node involvement compared with the other carcinomas. These 2 carcinomas showed a statistically significant difference with the other types of carcinomas in their Ki67 status, although higher levels of Ki67 indicate the invasive nature of the tumor and entail a worse prognosis. A significant relationship was also found between Ki67 and tumor grade, as tumors with higher grades have higher levels of cell proliferation and are consequently more invasive than the other grades; these findings were consistent with the findings of other studies.^27,28^ In the present study, tumors with higher Ki67 expression showed a higher HER2/neu overexpression as well; this correlation may be used to predict the biological behavior of breast cancer. This study also showed that Ki67 expression decreased as the patient's age increased, demonstrating that Ki67 expression is higher and the tumor is more invasive in younger patients. This result was consistent with the results obtained by Vasseur et al,^27^ who found Ki67 expression to be higher in younger patients compared with older patients. Although no significant relationships were found between the level of Ki67 expression and lymph node involvement in the present study, a positive relationship was observed between the mean level of Ki67 expression and involved lymph nodes. This result indicates that higher percentages of Ki67 expression result in more involved lymph nodes, making lymph node involvement a key issue to be considered in the case of Ki67-positive cells. Ki67 expression thus affects the prognosis of breast cancer along with other factors, including the size and grade of tumor; however, it is not significantly effective in and by itself. The last potential predictive/prognostic parameter examined in the present study was p53 mutation status. Various reports have outlined the role of p53 in the progression of breast carcinoma.^28^ In our study, although mucinous tumors had the highest p53 mutation, no statistically significant differences were observed between the different types of carcinomas in this regard. However, p53 mutation increased significantly with the grade of the tumor, as the highest p53 mutation was observed in grade 3 tumors. The pairwise comparisons also showed no significant relationships between p53 status and lymph node involvement and HER2/neu overexpression. On the basis of the calculated correlation coefficient, the size of tumor and age did not significantly correlate with p53 mutation; however, p53 showed an inverse correlation with age. Nevertheless, Kim et al^28^ concluded that with the increase in p53 mutation, tumors become more invasive and p53 mutation should be considered a negative predictor, yet there are still ambiguities in the routine evaluation of this factor in the different types of breast cancer. Literature review revealed that the Luminal A subtype was the most frequent subtype, and HER2 type with Basal-like type was the least; however, our study was not in line with these previous reports.^29,30^ Recent studies have focused on molecular subtypes of breast carcinomas, and hope to be useful in management and treatment of these lesions.^30,31^ In previous studies, the Luminal A group correlated with low-grade tumors and had better prognosis compared with others; however, in the present study no significant correlation was obtained between molecular subtype and grade of tumors.^30,31^

## CONCLUSION

Breast cancer patients with higher Ki67 expression showed more HER2/neu overexpression, and these cancers may be identified as aggressive tumors. Moreover, in young patients with breast carcinoma, the rates of Ki67 with overexpression of HER2/neu and p53 mutation were higher, and it is shown to be indicative of a more invasive tumor and a higher frequency of metastasis. However, p53 mutation was seen with higher tumor grades, but no significant correlation was obtained between its status and lymph node involvement, so its role in the progression of tumors remains unclear.
